# Preservation of the Foveal Avascular Zone in Achromatopsia Despite the Absence of a Fully Formed Pit

**DOI:** 10.1167/iovs.61.10.52

**Published:** 2020-08-31

**Authors:** Rachel E. Linderman, Michalis Georgiou, Erica N. Woertz, Jenna A. Cava, Katie M. Litts, Sergey Tarima, Ranjan Rajendram, Jan M. Provis, Michel Michaelides, Joseph Carroll

**Affiliations:** 1 Cell Biology, Neurobiology & Anatomy, Medical College of Wisconsin, Milwaukee, Wisconsin, United States; 2 Institute of Ophthalmology, University College London, London, United Kingdom; 3 Moorfields Eye Hospital NHS Foundation Trust, London, United Kingdom; 4 Ophthalmology & Visual Sciences, Medical College of Wisconsin, Milwaukee, Wisconsin, United States; 5 Division of Biostatistics, Institute for Health and Equity, Medical College of Wisconsin, Milwaukee, Wisconsin, United States; 6 The John Curtin School of Medical Research, The Australian National University, Canberra, Australian Capital Territory, Australia; 7 The ANU Medical School, The Australian National University, Canberra, Australian Capital Territory, Australia

**Keywords:** foveal avascular zone, achromatopsia, retinal development, optical coherence tomography angiography

## Abstract

**Purpose:**

To examine the foveal avascular zone (FAZ) in patients with congenital achromatopsia (ACHM).

**Methods:**

Forty-two patients with genetically confirmed ACHM were imaged either with Optovue's AngioVue system or Zeiss's Plex Elite 9000, and the presence or absence of a FAZ was determined. For images where a FAZ was present and could be confidently segmented, FAZ area, circularity index, and roundness were measured and compared with previously published normative values. Structural optical coherence tomography images were acquired to assess the degree of foveal hypoplasia (number and thickness of inner retinal layers present at the fovea).

**Results:**

A FAZ was present in 31 of 42 patients imaged (74%), although no determination could be made for 11 patients due to poor image quality (26%). The mean ± SD FAZ area for the ACHM retina was 0.281 ± 0.112 mm^2^, which was not significantly different from the previously published normative values (*P* = 0.94). However, their FAZs had decreased circularity (*P* < 0.0001) and decreased roundness (*P* < 0.0001) compared to the normative cohort. In the patients with ACHM examined here, the FAZ area decreased as the number and thickness of the retained inner retinal layers increased.

**Conclusions:**

Our data demonstrate that despite the presence of foveal hypoplasia, patients with ACHM can have a FAZ. This is distinct from other conditions associated with foveal hypoplasia, which generally show an absence of the FAZ. In ACHM, FAZ formation does not appear to be sufficient for complete pit formation, contrary to some models of foveal development.

The human fovea is a highly specialized region of the retina that underpins our high-acuity vision and color discrimination.^1^^,^^2^ The foveal region is characterized by an avascular zone (aka, the foveal avascular zone, or FAZ) and an absence of the inner retinal layers. While the fovea occupies only about 0.02% of the retinal surface area, some 40% of primary visual cortex (V1) receives input from the fovea.^3^ This is made possible by the high density of cone photoreceptors,^4^ the specialized “private line” circuitry between cones and midget ganglion cells,^5^^,^^6^ and increased cortical sampling of foveal inputs in V1.^7^^,^^8^ Given that foveal structure and function is affected in a wide range of retinal diseases that lead to severe visual impairment,^9^^–^^13^ there is significant interest in understanding how the fovea develops, especially the relationship between different foveal specializations.

Histologic studies have shown that a FAZ is defined before the pit begins to form.^14^ Additionally, in vivo studies using optical coherence tomography (OCT) have demonstrated a positive correlation between the size of the FAZ and the size of the pit in control populations.^15^^–^^18^ Moreover, patients with albinism lack both a FAZ and complete formation of the pit (i.e., foveal hypoplasia).^11^^,^^19^ Taken together, these data support current models of foveal development that propose the FAZ as a prerequisite for the formation of the pit.^1^^,^^20^ However, recent data have shown the presence of foveal microvessels (absence of a normal FAZ) in individuals with a normal-appearing pit,^21^^–^^27^ rather than overt foveal hypoplasia as might be expected. As foveal hypoplasia is present in a wide range of diseases (and even in normally sighted individuals),^11^^,^^28^^–^^34^ studying the FAZ in these patients may provide further insight into how these foveal specializations are related. Such data would be valuable in advancing our understanding of foveal development.

Congenital achromatopsia (ACHM) is an autosomal recessive disease characterized by almost complete loss of cone photoreceptor function.^35^ Symptoms of this disorder include nystagmus, low-acuity vision, reduced or absent color discrimination, and photophobia. These patients have a varying amount of inner retina present at the fovea, ranging from severe foveal hypoplasia to normal (complete) displacement of inner retinal layers at the fovea,^36^ although there have been no studies of the FAZ in these patients. Here we report the discovery of normal-sized FAZs in patients with ACHM, most of whom have persistent inner retinal layers at the fovea. Our data demonstrate that the formation of a FAZ is not sufficient for normal pit formation in ACHM.

## Materials and Methods

### Patients

The study followed the tenets of the Declaration of Helsinki and was approved by the institutional review boards at the Medical College of Wisconsin and Moorfields Eye Hospital. Informed consent was obtained from all patients after the nature and possible consequences of the study were explained. Forty-two patients with genetically confirmed ACHM were enrolled for imaging (Supplementary Table S1). The patients with ACHM imaged at the Medical College of Wisconsin (*n* = 27) were screened for a history of premature birth. In addition, a 26-year-old woman with no known vision pathology and no history of premature birth was recruited for this study for illustrative purposes. Axial length measurements were obtained from all individuals using an IOLMaster (Carl Zeiss Meditec, Dublin, CA, USA). When necessary, one drop each of tropicamide (1%) and of phenylephrine hydrochloride (2.5%) were instilled into the eye for pupillary dilation and cycloplegia.

### Spectral Domain OCT (SD-OCT) Imaging

High-resolution structural images of the macula were acquired using a Bioptigen Envisu R2200 SD-OCT system (Leica Microsystems, Wetzlar, Germany) or Spectralis SD-OCT system (Heidelberg Engineering, Heidelberg, Germany). For patients imaged using the Bioptigen (*n* = 35), high-density line scans (1000 A-scans/B-scans, 80 repeated B-scans) and nominal 7-mm volume scans (750 A-scans/B-scans, 250 B-scans) were acquired at the fovea. Line scans were registered based on the presence of the foveal reflex and averaged to reduce speckle noise in the image.^37^ For patients imaged using the Spectralis (*n* = 7), 20˚ or 30˚ OCT volume or line scans were acquired. To ensure the scans used for analysis were at the foveal center, volume scans were manually inspected by one observer (R.E.L.) to confirm the location of the fovea. One observer (K.M.L.) graded all foveal line scans for disruption of the ellipsoid zone (i.e., photoreceptor band) using a previously established grading system.^36^^,^^38^ Three graders (R.E.L., E.N.W., and J.C.) reviewed all the foveal line scans to determine if there were retained inner retinal layers and (if present) which layers were retained. Graders were masked as to whether a given subject had a FAZ, and a consensus approach was used in cases of disagreement. Additionally, using logarithmic grayscale images and custom software (OCT Reflectivity Analytics, Milwaukee, WI, USA), longitudinal reflectance profiles were used to measure the thickness of the inner retinal layers and the outer nuclear layer at the fovea for each subject.^39^ Two repeated thickness measurements were made for each layer and the averaged values were used for subsequent analyses.

### OCT Angiography (OCT-A) Imaging and Analysis

Patients were imaged either using the AngioVue system (Optovue, Inc., Fremont, CA, USA) at the Medical College of Wisconsin or the Plex Elite 9000 (Carl Zeiss; Meditec, Dublin, CA, USA) at Moorfields Eye Hospital. When possible, both eyes were imaged, and the eye with the better image quality was chosen for analysis. The remaining patients had only one eye dilated for other imaging, so that eye was used for this study. For the AngioVue system, two volumes consisting of 304 B-scans at 304 A-scans/B-scans were acquired, centered on the fovea, with a nominal size of 3 × 3 mm. One volume had a horizontal fast scanning axis, while the other volume had a vertical fast scanning axis. The two volumes were registered (AngioVue software version: 2017.1.0.151) to create a single volume with reduced motion artifact and increased signal-to-noise ratio (SNR). From each volume, the full-thickness angiogram was extracted by integrating motion contrast data from the inner limiting membrane (ILM) to 9 µm above the outer plexiform layer (OPL). Three to seven volumes were obtained per patient based image quality. Where possible, three to five volumes were then averaged together as previously described^40^^,^^41^ to further increase image SNR. The averaged image was used for FAZ analyses.

For the Plex Elite 9000, one volume consisting of 1024 B-scans and 350 A-scans/B-scans was acquired with a horizontal fast scanning axis. The nominal size was also 3 × 3 mm. The superficial layer, measuring from the ILM to the outer boundary of the inner plexiform layer (IPL), was extracted and single frames were used for FAZ analyses. The real scale of each image (µm/pixel) was calculated by multiplying the nominal image scale by the ratio of the patient's axial length to the axial length assumed by the OCT-A device (Zeiss = 24.46 mm, Optovue = 23.95 mm).

**Figure 1.**
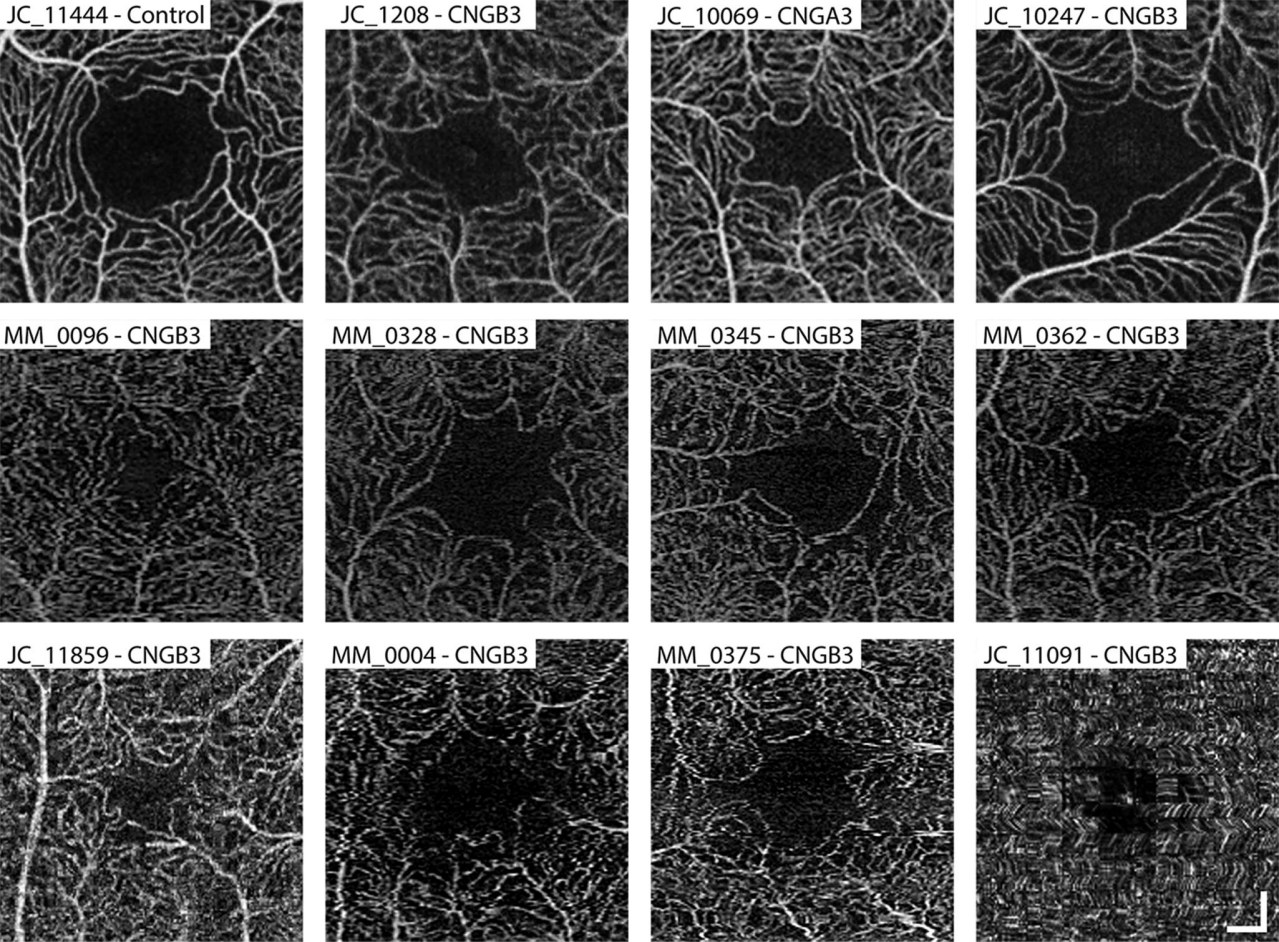
Representative OCT-A images used to subjectively decide if a FAZ is present and, if present, analyzable. An OCT-A scan from the right eye of a 26-year-old female subject with normal vision is shown for reference (*top left*). This normal-appearing FAZ has an area of 0.389 mm^2^, a circularity index of 0.593, and a roundness of 0.852. The remaining panels in the *top row* are examples of patients imaged using the Optovue AngioVue system with multiple frames averaged together. The *second row* includes single-frame images from patients imaged using the Zeiss Plex Elite 9000. All seven patients in the *top two rows* were determined to have a present and analyzable FAZ. The first three patients in the *bottom row* were all graded as having a FAZ, but distortions at the fovea make segmentation and measurement of the FAZ unreliable. The subject in the *bottom right* had poor image quality that made it not possible to determine whether a FAZ was present. *Scale bars*: 200 µm.

Determination of whether a FAZ was present was done by a single observer (R.E.L.) with three different possibilities: FAZ present, FAZ not present, and FAZ presence not determined due to poor image quality (Fig. 1). For the images in which a FAZ was present, a single observer (R.E.L.) subjectively determined if the image was of sufficient quality for segmentation (i.e., minimal motion artifacts around the FAZ, good vessel contrast, no motion artifacts, etc.). If the image quality was deemed sufficient, the FAZ for each patient was segmented by the same observer using ImageJ software's multipoint tool (National Institutes of Health, Bethesda, MD, USA).^42^ The segmentation coordinates were entered into a custom MATLAB (MathWorks, Inc., Natick, MA, USA) script based on previous work in our lab.^43^^,^^44^ The area of the FAZ (in pixels) was computed using the *poly2area* function. The perimeter of the FAZ (in pixels) was calculating by simply summing the distances between each pair of neighboring segmentation coordinates. The area and perimeter were both converted into retinal units (millimeters) by multiplying by the real scale of that image (described above). Circularity is a unitless metric that is defined by the following equation:

$$Circularity=4\pi *\left(\frac{FAZ\;Area}{FAZ\;Perimeter^{2}}\right)$$

Additionally, using the MATLAB function *poly2mask,* a mask was produced from the segmentation coordinates and *regionprops* was used to derive an ellipse with the same normalized second central moment (variance) as the mask. The normalized second central moment is calculated as follows:

$$\begin{array}{c} \begin{array}{c} Normalized\;2nd\;Central\;Moment=\;\frac{\sum_{i=1}^{n}{\left(\sqrt{{\left(x_{i}-x_{c}\right)}^{2}+\;{\left(y_{i}-\;y_{c}\right)}^{2}}\right)}^{2}}{Area} \end{array} \end{array}$$

where the centroid of the FAZ mask (or ellipse) is *x_c_,y_c_* and *x_i_,y_i_* represents every point within the FAZ mask (or ellipse). The major axis (longest diameter, pixels) and the minor axis (shortest diameter, pixels) of the resulting ellipse were extracted. These values were again converted into retinal units (millimeters) by multiplying by the real scale of the image. Roundness is a unitless metric that is defined by the following equation:

$$Roundness=\left(\frac{4\;*\;FAZ\;Area}{\pi \;*\;Major\;Axis^{2}}\right)=\frac{Minor\;Axis}{Major\;Axis}$$

All statistical analyses were conducted using Prism v8.2.1 (GraphPad Software, La Jolla, CA, USA). Specific statistical tests are reported alongside the individual results. The choice of parametric versus nonparametric test was based on an analysis of normality for each group using the Shapiro-Wilk test (using *P* > 0.05 as the criterion).

## Results

The demographics for the 42 patients with ACHM recruited for this study are given in Supplementary Table S1, 25 of whom have been previously reported in other studies (Supplementary Table S2).^36^^,^^38^^,^^45^^–^^49^ There were 19 men and 23 women, with a mean ± SD age of 29.2 ± 13.4 years. None of the 27 patients with ACHM imaged on the AngioVue system reported a history of premature birth. A FAZ was present in 31 of 42 individuals imaged (74%), and no determination could be made for 11 patients due to poor image quality (26%). Example OCT-A images, including those for which no determination could be made, are shown in Figure 1. Of the 31 patients who were judged to have a FAZ, 24 (77%) had minimal motion artifacts around the FAZ, which allowed for confident segmentation and derivation of quantitative FAZ metrics. No significant difference in FAZ area, circularity, and roundness was observed between the devices used here (Table 1; *P* = 0.91, *P* = 0.93, and *P* = 0.72, respectively). As such, we treated the patients with ACHM as a single group for subsequent analyses.

**Table 1.** FAZ Metrics Summary of the Subject Population

		FAZ Metrics^*^^,^^†^		
OCTA Device	Number of Eyes	Area (mm^2^)	Circularity Index	Roundness
AngioVue	16	0.279 ± 0.120	0.502 ± 0.098	0.756 ± 0.073
Plex Elite 9000	8	0.285 ± 0.100	0.498 ± 0.088	0.766 ± 0.061
		*P* = 0.91^‡^	*P* = 0.93^‡^	*P* = 0.72^‡^

* All data are listed as mean ± standard deviation.

† All data passed Shapiro-Wilk normality test (*P* > 0.05).

‡ Unpaired *t*-test.

In order to evaluate whether the FAZ morphometry observed in our patients with ACHM differed from normal, we compared their data to a group of 175 previously published patients with no vision pathology.^50^ The mean ± SD FAZ area for the control group was 0.280 ± 0.101 mm^2^, which was not significantly different from the patients with ACHM (0.281 ± 0.112 mm^2^; *P* = 0.94, Mann-Whitney *U* test). In contrast, the circularity index of the FAZ in the control group (mean ± SD = 0.682 ± 0.106) was significantly greater than that for the patients with ACHM (0.501 ± 0.093; *P* < 0.0001, unpaired *t*-test). Likewise, the roundness of the FAZ in the control group (mean ± SD = 0.860 ± 0.068) was significantly greater than that for the patients with ACHM (0.759 ± 0.068; *P* < 0.0001, Mann-Whitney *U* test). Additional normative FAZ morphometry data from other published studies are provided in Table 2 for reference, which are consistent with the FAZ in ACHM being normal in area but more irregular in overall contour.

**Table 2.** Summary of Previous OCT-A Studies Assessing FAZ Area, Circularity Index, and Roundness in Patients Without Disease

			FAZ Metrics^*^		
Study	OCTA Device	Number of Eyes	Area (mm^2^)^†^	Circularity Index	Roundness
Yasin Alibhai et al^65^	AngioVue	40	0.33 ± 0.15	0.73 ± 0.19^‡^	ND
Arya et al^66^	Angioplex	8	0.333 ± 0.063	ND	ND
	AngioVue		0.312 ± 0.073	ND	ND
	Plex Elite 9000		0.305 ± 0.071	ND	ND
Borrelli et al^67^	Angiovue	77	0.261 ± 0.149	ND	ND
Chen et al^68^	Angiovue	50	0.233 ± 0.108^§^	ND	ND
Choi et al^69^	Angioplex	52	0.35 ± 0.11	0.81 ± 0.07	ND
Corvi et al^70^	Angioplex	36	0.232 ± 0.004	ND	ND
	AngioVue		0.2221 ± 0.1002	ND	ND
	Plex Elite 9000		0.2250 ± 0.1004	ND	ND
Durbin et al^71^	Angioplex	50	0.25 ± 0.10	0.82 ± 0.06	ND
Hsieh et al^72^	AngioVue	22	0.35 ± 0.08	0.74 ± 0.11^‡^	ND
Inanc et al^73^	AngioVue	57	0.27 ± 0.13	0.94 ± 0.13^‡^	ND
Ishii et al^74^	Plex Elite 9000	40	0.244 ± 0.81	ND	ND
Lin et al^75^	Angioplex	35	0.255 ± 0.112	0.64 ± 0.14	ND
Linderman et al^50^^∥^	AngioVue	175	0.280 ± 0.101^§^	0.682 ± 0.106	0.860 ± 0.068
Sacconi et al^76^	Plex Elite 9000	32	0.199 ± 0.100	ND	ND
Shiihara et al^77^	AngioVue	70	0.329 ± 0.115	0.769 ± 0.064	0.878 ± 0.071

ND, not determined.

* All data are listed as mean ± standard deviation.

† Significant digits are listed as reported in the original study.

‡ Study originally reported circularity as acircularity. Acircularity was converted to circularity using the following equation: *Circularity* =  1/*Acircularity*^2^.

^§^Study corrected FAZ area for ocular magnification.

∥ The right eye of the original data was reprocessed with the same code used in the current study.

**Figure 2.**
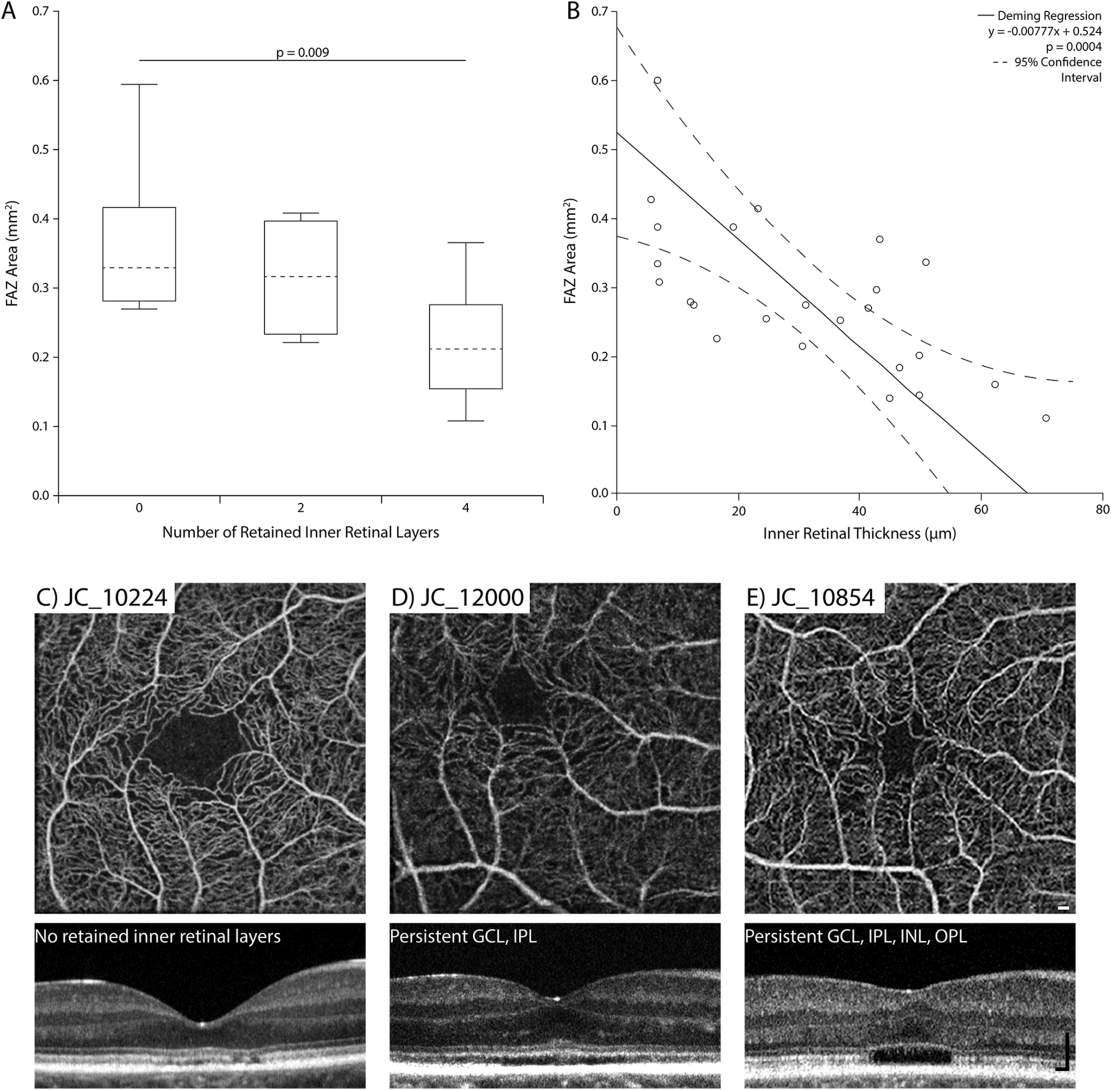
Relationship between retained inner retinal layers at the fovea and FAZ morphometry in patients with ACHM. (**A**) As shown in the box-and-whiskers plot (*dashed line*: median; *box limits*: 25th–75th percentiles; *whiskers*: minimum and maximum), there was a significant difference in FAZ area between patients with different numbers of retained inner retinal layers (one-way ANOVA, *P* = 0.01). Further analysis showed the only significant difference between groups occurred between patients with no retained inner retinal layers (0) and those with all four layers retained (Tukey's multiple comparison test, *P* = 0.009). (**B**) There was a significant negative relationship between the inner retinal layer thickness and FAZ area (Deming regression, *y* = –0.00777*x* + 0.524, *P* = 0.004). The 95% confidence intervals (*dashed lines*) were calculated using the jackknife method. (**C**) This subject has normal inner retinal layer appearance at the fovea (zero layers retained). Inner retinal thickness = 7.17 µm, FAZ area = 0.384 mm^2^, circularity index = 0.524, and roundness = 0.731. (**D**) This subject has two layers retained (GCL and IPL). Inner retinal thickness = 16.73 µm, FAZ area = 0.220 mm^2^, circularity index = 0.560, and roundness = 0.786. (**E**) This subject has four layers retained (GCL, IPL, INL, and OPL). Inner retinal thickness = 62.74 µm, FAZ area = 0.155 mm^2^, circularity index = 0.383, and roundness = 0.597. Note that this subject has a disrupted ellipsoid zone (aka, a hyporeflective zone) at the fovea, which is commonly seen in patients with ACHM. *Scale bars*: 100 µm.

We sought to determine if there was a relationship between the size of the FAZ and the foveal retinal layers. Of the 31 patients in whom a FAZ was determined to be present, 22 (71%) had retained inner retinal layers at the fovea. Of these patients, 17 (77%) had retention of all inner retinal layers (ganglion cell layer (GCL), IPL, INL, and OPL) and 5 (23%) had retention of GCL and IPL, but no inner nuclear layer (INL) and OPL. The remaining 9 patients (29%) appeared to have complete displacement of the inner retinal layers at the fovea. As shown in Figure 2A, FAZ area decreased as the number of retained inner retinal layers increased (one-way ANOVA, *P* = 0.01), although there was significant overlap between groups. FAZ area was significantly smaller in patients with four retained inner retinal layers compared to those with no observable retained inner retinal layers (Tukey's multiple comparison test, *P* = 0.009). However, there was no significant difference in FAZ area between patients with no observable retained inner retinal layers and those with two retained inner retinal layers (Tukey's multiple comparison test, *P* = 0.64) or between patients with two retained inner retinal layers and those with four retained inner retinal layers (Tukey's multiple comparison test, *P* = 0.22). Additionally, as the thickness of the retained inner retinal layers increased, the FAZ area decreased (Deming regression, *P* = 0.0004, Fig. 2B). Images from patients demonstrating this trend are shown in Figures 2C–2E. In contrast to FAZ area, there was no significant relationship between inner retinal layer thickness and either FAZ circularity index (Deming regression, *P* = 0.27) or FAZ roundness (Deming regression, *P* = 0.44), as shown in Supplementary Figure S1, although there was small but significant relationship between outer nuclear layer thickness and FAZ area (Supplementary Figure S2; Deming regression, *P* = 0.05). Finally, there was no association between FAZ area and the severity of the ellipsoid zone disruption (Kruskal-Wallis test, *P* = 0.14).

Since we could not determine the presence of the FAZ in all patients, we wanted to examine whether a selection bias may be influencing our results relating FAZ area with foveal inner retinal lamination. There was no difference in inner retinal thickness (unpaired *t*-test, *P* = 0.71) or age (Mann-Whitney *U* test, *P* = 0.74) between patients with a FAZ and patients for whom the presence of a FAZ could not be determined. Likewise, we saw no difference in the distribution of mutation (Fisher’ exact test, *P* = 0.64), sex (Fisher's exact test, *P* = 0.73), ellipsoid zone disruption (chi-square test, *P* = 0.66), or the number of persistent inner retinal layers at the fovea (chi-square test, *P* = 0.29). These findings suggest that our observed relationship between FAZ area and inner retinal layer thickness likely holds for all patients with *CNGA3-* or *CNGB3-*associated ACHM.

## Discussion

As OCT and OCT-A imaging have become standard for both clinical care in ophthalmology and vision research, the relationship between the fovea and the FAZ has been scrutinized in an attempt to understand how the fovea develops. Despite a number of studies,^11^^,^^16^^,^^17^^,^^20^ the relationship between these specializations remains unclear. Studies suggest that the FAZ is delineated prior to any pit formation; however, the mechanism for how the pit is formed as well as the potential role of the FAZ in this process remains uncertain. One theory suggests that the pit is passively created due to an increased elasticity of the incipient fovea (due to a lack of vasculature in the region), an increase in interocular pressure, and normal eye growth.^1^ Another theory suggests the metabolic demands of the foveal region causes a hypoxic environment, which in turn causes the displacement of the inner retinal layers.^2^ Here we show, for the first time, FAZ areas within the normal anatomical range in patients with foveal hypoplasia. It is important to consider that 9 of the 31 patients with ACHM for whom we measured the FAZ had no inner retinal layers present at their fovea. In these patients, the presence of a FAZ is not at all surprising. Even if we remove these 9 patients from the analysis, the average FAZ area (0.245 mm^2^, *n* = 22) was not significantly different from the controls (Mann-Whitney *U* test, *P* = 0.157). However, we do see a negative relationship between the size of the FAZ present and the residual inner retina at the fovea, which supports a facilitatory role of the FAZ in the process of pit development as previously predicted from finite element modeling.^51^ Taken together, our data clearly demonstrate that a normal-sized FAZ alone is not sufficient to effect complete pit formation in ACHM.

Further insight on foveal development comes from comparing our findings in ACHM to findings in albinism. Patients with albinism have a similar persistence of inner retinal layers across the fovea, but the sparse data available to date have shown that they lack a FAZ (Phillips E, et al. *IOVS* 2016;57:ARVO E-Abstract 5461).^19^^,^^52^^,^^53^ While patients with albinism have reduced peak foveal cone densities compared to control populations,^11^ these cones are nonetheless functional. In contrast, patients with ACHM have severely diminished to no cone function.^54^ This, in turn, may lead to reduced activity of the midget ganglion cells in the foveal region in patients with ACHM, which could reduce the overall metabolic demands of this region. If true, this would support a role for cone pathway activity in the process of foveal development and offers a possible area for further investigation in nonhuman primate models.

Our findings in ACHM (normally sized FAZs in the presence of foveal hypoplasia) are in stark contrast to those in other conditions associated with foveal hypoplasia.^12^^,^^13^^,^^34^^,^^55^^–^^57^ For example, Yanni et al.^57^ observed a FAZ in preterm children, although again about half the area of that seen in full-term children. They used an en face OCT method that examined the deep capillary plexus, so they would have missed any persistent capillaries in the superficial plexus. Falavarjani et al.^12^ found that a distinct FAZ was absent in 42.8% of eyes in preterm children. When a FAZ was present, it was accompanied by an absence of inner retinal layers at the fovea and was considerably smaller than normal and appeared as a fragmented structure opposed to a single avascular area. This is similar to that reported by Miki et al.,^56^ who observed small (average = 0.14 mm^2^), fragmented FAZ-like structures in patients with a history of laser treatment for retinopathy of prematurity. Another study of children with a history of treatment for retinopathy of prematurity (laser or intravitreal ranibizumab) also reported very small and fragmented FAZ-like structures, often accompanied by an absence of persistent inner retinal layers at the fovea.^55^ Not only are the FAZs reported in our study not significantly different from normal in area, but there was only one subject with ACHM who had a fragmented FAZ, assessed using our recently described objective and automated method.^58^ It would be interesting to reexamine OCT-A images from patients with foveal hypoplasia and exclude those with a fragmented FAZ before quantitative analyses.

Our study has a number of important limitations. Most notably, only individuals with *CNGA3* or *CNGB3* genotypes were included. While there are reported differences in cone structure in the *GNAT2*,^59^^,^^60^ *ATF6*,^61^ and *PDE6C* forms of ACHM,^62^ it is unknown whether a FAZ is present in individuals with ACHM caused by these genotypes. Additionally, there are reports of residual cone function in patients with ACHM,^63^ and patients with *GNAT2*-associated ACHM have been reported to have cone-mediated visual function at high radiances (although only at low temporal frequencies).^64^ While we did not assess cone function in the patients imaged in the present study, it would be valuable to examine the FAZ in patients with ACHM who have clearly demonstrable cone function as this would advance our understanding of how metabolic demands may impact FAZ and pit formation. Another limitation is that we did not employ an age-, race-, or sex-matched control group. These variables have been shown to affect the FAZ morphometry, although the numerous studies listed in Table 2 provide confidence that the FAZ area observed in our ACHM cohort is well within published normative ranges. A final limitation relates to the interpretation of our key observation (presence of a normal-sized FAZ in ACHM). While our youngest subject was 10 years old, the FAZ normally forms prenatally.^1^ Thus, we cannot rule out that there was no FAZ at birth in any of our patients and that vascular remodeling (such as that occurs in diabetic retinopathy^9^) occurred postnatally to effectively create a FAZ. The normal area of the FAZ in ACHM would argue against this, although the decreased circularity/roundness of the FAZ in ACHM could be considered consistent with this alternate interpretation. However, the nystagmus present in patients with ACHM can result in distortions in the OCTA image (even in images with high SNR), which would affect the measurements of the circularity/roundness of the FAZ. Thus, we believe the simplest explanation is that the FAZ developed normally but the pit did not fully form in all patients.

In conclusion, patients with ACHM-associated foveal hypoplasia have a FAZ that suggests that the FAZ is necessary but not sufficient for normal pit formation for these patients. These data should help in further constraining comprehensive models of foveal development as well as shed additional light on the complex relationship between the different anatomic specializations of the human fovea.

## Supplementary Material

Supplement 1

Supplement 2

Supplement 3

Supplement 4
