# Supplementary material for: Preservation of the Foveal Avascular Zone in Achromatopsia Despite the Absence of a Fully Formed Pit
Source: Invest Ophthalmol Vis Sci. 2020 Aug 31;61(10):52. doi: 10.1167/iovs.61.10.52 (PMC7463179; doi:10.1167/iovs.61.10.52)
Supplement: Supplement 1 [file iovs-61-10-52_s001.pdf]

Supplementary Table 1. Demographics and data summary for all subjects used

Subject	Sex	Age (years)	Genotype	Eye*	OCT Grade†	OCT-A Device‡	FAZ Present§	FAZ Analyzable‖
JC_11444	F	26	None (Control)	OD	NA	AngioVue	Yes	Yes
TC_11579	M	10	B3	OD	1	AngioVue	Yes	Yes
JC_10249	M	19	B3	OD	1	AngioVue	Yes	Yes
JC_11326	F	25	B3	OS	1	AngioVue	Yes	Yes
MM_0096	F	31	B3	OD	1	PE 9000	Yes	Yes
JC_11664	M	36	B3	OD	1	AngioVue	Yes	Yes
JC_12000	F	57	B3	OD	1	AngioVue	Yes	Yes
JC_11623	M	15	B3	OD	2	AngioVue	Yes	Yes
MM_0362	F	16	B3	OD	2	PE 9000	Yes	Yes
MM_0162	F	19	B3	OD	2	PE 9000	Yes	Yes
JC_1208	M	21	B3	OD	2	AngioVue	Yes	Yes
JC_10069	M	22	A3	OD	2	AngioVue	Yes	Yes
MM_0328	F	25	B3	OD	2	PE 9000	Yes	Yes
JC_10853	F	31	B3	OD	2	AngioVue	Yes	Yes
JC_11871	M	31	B3	OD	2	AngioVue	Yes	Yes
JC_10494	M	31	B3	OD	2	AngioVue	Yes	Yes
JC_11860	M	34	B3	OD	2	AngioVue	Yes	Yes
MM_0345	F	34	B3	OD	2	PE 9000	Yes	Yes
MM_0359	F	35	B3	OD	2	PE 9000	Yes	Yes
JC_10224	F	41	B3	OD	2	AngioVue	Yes	Yes
JC_11990	F	21	B3	OD	4	AngioVue	Yes	Yes
MM_0171	M	23	A3	OD	4	PE 9000	Yes	Yes
JC_10247	M	28	B3	OS	4	AngioVue	Yes	Yes
JC_10854	F	34	B3	OD	4	AngioVue	Yes	Yes
MM_0014	F	35	A3	OD	4	PE 9000	Yes	Yes
MM_0375	F	22	B3	OD	1	PE 9000	Yes	No
MM_0064	F	25	A3	OD	2	PE 9000	Yes	No
JC_10968	M	46	B3	OD	2	AngioVue	Yes	No
JC_11859	F	23	B3	OD	3	AngioVue	Yes	No
JC_10151	F	14	B3	OD	4	AngioVue	Yes	No
JC_0686	F	55	B3	OD	4	AngioVue	Yes	No
MM_0004	M	30	B3	OD	5	PE 9000	Yes	No
JC_10250	M	21	B3	OS	1	AngioVue	ND	NA
JC_11091	F	33	B3	OD	1	AngioVue	ND	NA
KS_10088	F	68	A3	OD	1	AngioVue	ND	NA
MM_0099	M	13	B3	OD	2	PE 9000	ND	NA
MM_0120	M	13	B3	OD	2	PE 9000	ND	NA
MM_0072	F	14	B3	OD	2	PE 9000	ND	NA
JC_12001	F	61	B3	OD	2	AngioVue	ND	NA
MM_0387	M	19	A3	OD	4	PE 9000	ND	NA
JC_10167	F	20	B3	OD	4	AngioVue	ND	NA
JC_10196	F	36	B3	OD	4	AngioVue	ND	NA
JC_10226	F	41	B3	OD	5	AngioVue	ND	NA

*OD = right; OS = left

†OCT grade refers to the level of disruption of the ellipsoid zone, defined by Sundaram *et al*. (2014)

‡PE 9000 = Plex Elite 9000

§ND = not determined

‖NA = not applicable
