# Supplementary material for: Preservation of the Foveal Avascular Zone in Achromatopsia Despite the Absence of a Fully Formed Pit
Source: Invest Ophthalmol Vis Sci. 2020 Aug 31;61(10):52. doi: 10.1167/iovs.61.10.52 (PMC7463179; doi:10.1167/iovs.61.10.52)
Supplement: Supplement 2 [file iovs-61-10-52_s002.pdf]

Supplementary Table 2. Previous Reporting of ACHM Subjects

Subject ID in Current Study	Sundaram et al (2014) & Aboshiha et al (2014)	Dubis et al (2014)	Scoles et al (2014)	Langlo et al (2016)	Langlo et al (2017)	Hirji et al (2018)	Georgiou et al (2019)	Litts et al (2020)	Georgiou et al (2020)
TC_11579									
JC_10249				UFC-009	UFC-009				
JC_11326									
MM_0096						34			
JC_11664									
JC_12000									
JC_11623									
MM_0362									
MM_0162	23					24		same ID	same ID
JC_1208		same ID		PCI-009	PCI-009			same ID	
JC_10069			same ID				same ID	same ID	same ID
MM_0328								same ID	same ID
JC_10853				PCI-032				same ID	
JC_11871									
JC_10494									
JC_11860									
MM_0345								same ID	same ID
MM_0359									
JC_10224				PCI-021	PCI-021			same ID	
JC_11990									
MM_0171	5					5	same ID		same ID
JC_10247				UFC-001	UFC-001			same ID	
JC_10854				PCI-033				same ID	
MM_0014	12					12	same ID		
MM_0375									
MM_0064	6					6	same ID		same ID
JC_10968				PCI-034					
JC_11859									
JC_10151				PCI-006	PCI-006			same ID	
JC_0686									
MM_0004	28	same ID				28			
JC_10250				UFC-008	UFC-008				
JC_11091									
KS_10088			same ID				same ID		same ID
MM_0099									
MM_0120						38			
MM_0072						40			
JC_12001									
MM_0387							same ID		
JC_10167				BPE-003	BPE-003			same ID	
JC_10196				UFC-002	UFC-002			same ID	
JC_10226				UFC-010					

References

Aboshiha J, Dubis AM, Cowing J, et al. A prospective longitudinal study of retinal structure and function in achromatopsia. Invest Ophthalmol Vis Sci 2014;55:5733-5743.

Dubis AM, Cooper RF, Aboshiha J, et al. Genotype-dependent variability in residual cone structure in achromatopsia: towards developing metrics for assessing cone health. Invest Ophthalmol Vis Sci 2014;55:7303-7311.

Georgiou M, Litts KM, Kalitzeos A, et al. Adaptive optics retinal imaging in CNGA3-associated achromatopsia: Retinal characterization, interocular symmetry, and intrafamilial variability. Invest Ophthalmol Vis Sci 2019;60:383-396.

Georgiou M, Litts KM, Singh N, Kane T, Patterson EJ, Hirji N, Kalitzeos A, Dubra A, Michaelides M, Carroll J. Intraobserver repeatability and interobserver reproducibility of foveal cone density measurements in CNGA3- and CNGB3-achromatopsia. Trans Vis Sci Tech. 2020;9:37.

Hirji N, Georgiou M, Kalitzeos A, et al. Longitudinal assessment of retinal structure in achromatopsia patients with long-term follow-up. Invest Ophthalmol Vis Sci 2018;59:5735-5744.

Langlo CS, Patterson EJ, Higgins BP, et al. Residual foveal cone structure in CNGB3-associated achromatopsia. Invest Ophthalmol Vis Sci 2016;57:3984-3995.

Langlo CS, Erker LR, Parker M, et al. Repeatability and longitudinal assessment of foveal cone structure in CNGB3-associated achromatopsia. Retina 2017;37:1956-1966.

Litts KM, Georgiou M, Langlo CS, et al. Interocular symmetry of foveal cone topography in congenital achromatopsia. Curr Eye Res 2020;1-8.

Scoles D, Sulai YN, Langlo CS, et al. In vivo imaging of human cone photoreceptor inner segments. Invest Ophthalmol Vis Sci 2014;55:4244-4251.

Sundaram V, Wilde C, Aboshiha J, et al. Retinal structure and function in achromatopsia: implications for gene therapy. Ophthalmology 2014;121:234-245.
