# Supplementary material for: Preservation of the Foveal Avascular Zone in Achromatopsia Despite the Absence of a Fully Formed Pit
Source: Invest Ophthalmol Vis Sci. 2020 Aug 31;61(10):52. doi: 10.1167/iovs.61.10.52 (PMC7463179; doi:10.1167/iovs.61.10.52)
Supplement: Supplement 3 [file iovs-61-10-52_s003.pdf]

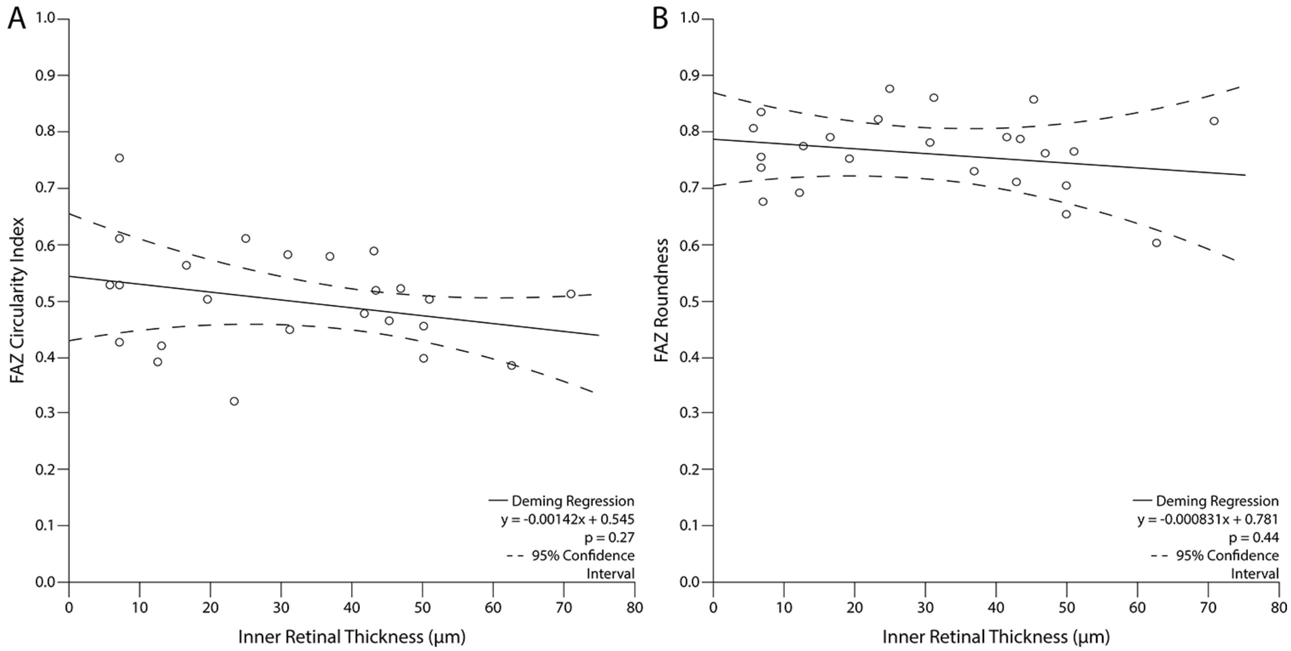

Supplementary Figure 1. Relationship between inner retinal layer thickness and FAZ shape in subjects with ACHM. **(A)** There was no significant relationship between inner retinal layer thickness and FAZ circularity index in subjects with ACHM (Deming regression: y = -0.000142x + 0.545, p = 0.27). **(B)** There was also no significant relationship between inner retinal layer thickness and FAZ roundness in subjects with ACHM (Deming regression: y = -0.000831x + 0.785, p = 0.44). 95% confidence intervals (dashed lines) were calculated using the jackknife method. See main manuscript text for definitions of FAZ circularity index and FAZ roundness.
