# Supplementary material for: Preservation of the Foveal Avascular Zone in Achromatopsia Despite the Absence of a Fully Formed Pit
Source: Invest Ophthalmol Vis Sci. 2020 Aug 31;61(10):52. doi: 10.1167/iovs.61.10.52 (PMC7463179; doi:10.1167/iovs.61.10.52)
Supplement: Supplement 4 [file iovs-61-10-52_s004.pdf]

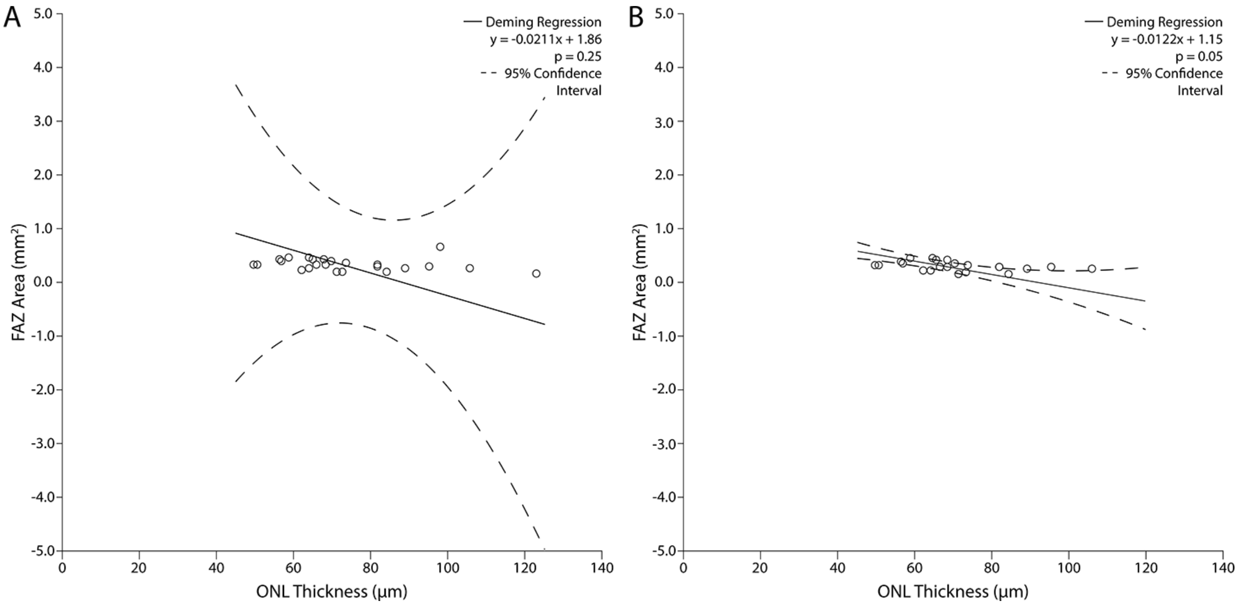

Supplementary Figure 2. Relationship between outer nuclear layer (ONL) thickness and FAZ area in subjects with ACHM. **(A)** There was no significant relationship between ONL layer thickness and FAZ area in subjects with ACHM (Deming regression: y = -0.0211x + 1.86, p = 0.25) when all data points were used. The wide confidence intervals were due to uncertainty in the predicted regression coefficients. **(B)** Plot of ONL thickness versus FAZ area with two outliers removed (we used the optimized residuals from the regression in panel A to identify outliers). The similar slope and y intercept did not change much (Deming regression, y = -0.0122x + 1.15), but the reduced uncertainty for the regression coefficients resulted in smaller confidence intervals and the relationship met statistical significance (p = 0.05). 95% confidence intervals (dashed lines) were calculated using the jackknife method.
